# A case report of ten-month-neglected anterior shoulder dislocation managed by open reduction combined with Latarjet procedure

**DOI:** 10.1016/j.ijscr.2019.03.008

**Published:** 2019-03-21

**Authors:** Andri Maruli Tua Lubis, Muhammad Rizqi Adhi Primaputra, Ismail H. Dilogo

**Affiliations:** Department of Orthopaedics and Traumatology, Cipto Mangunkusumo General Hospital/Faculty of Medicine Universitas Indonesia, Salemba Raya No. 6, Jakarta 10430, Indonesia

**Keywords:** Neglected shoulder dislocation, Open reduction, Latarjet procedure, Case report

## Abstract

**Introduction:**

Neglected shoulder dislocation is a rare case and may be accompanied by pathological changes in bony and soft tissue structure. Therefore, it requires extensive surgical procedure. Until now, there is no standard treatment protocol to overcome this case and it is still a challenging case.

**Presentation of case:**

We presented a 27-year-old male patient with deformity on his left shoulder. The patient has suffered a ten-month-neglected anterior shoulder dislocation with a Hill-Sachs lesion. The treatment was open reduction combined with Latarjet procedure. Evaluation of treatment was performed three months after surgery.

**Discussion:**

Management of a neglected shoulder dislocation, especially with signiﬁcant bony defects, is challenging and cannot be managed with standard surgical technique because of severe soft-tissue contracture and imbalance as well as bone deﬁciency. Chronic locked anterior shoulder dislocation is a difficult condition for both patient and clinician. In addition to that, the treatment results can be unsatisfactory. Latarjet procedure has been shown to be effective for the treatment of recurrent anterior shoulder dislocation with a large glenoid osseous defect which might justify the application of this procedure for the treatment of neglected anterior shoulder dislocation.

**Conclusion:**

Open reduction combined with Latarjet procedure performed for treatment of neglected anterior shoulder dislocation was found to have a high rate of successful in preventing further dislocation of the shoulder joint although the high risk of osteoarthritis of the shoulder joint can still persist.

## Highlights

• Neglected shoulder dislocation is relatively rare case but the management is challenging.
• Dislocated should be reduced as soon as possible to prevent complicated situation.
• Latarjet procedure is an effective treatment of choice for neglected shoulder dislocation.

## 1 Introduction

Shoulder joint is the most common dislocated joint [1,2]. Anterior dislocation of shoulder occurs more frequent, it accounts for 95% of all shoulder dislocation, rather than posterior dislocation [3,4]. A neglected shoulder dislocation is rare and may be accompanied by pathological changes in bony and soft tissue structures. Therefore, it requires extensive surgical procedure [5,6]. Until now, there is no standard treatment for this case and it is a difﬁcult problem for both patients and clinicians. We present a 27-year-old male who has suffered neglected anterior dislocation for ten months with a Hill-Sachs lesion. We managed this case by open reduction and Latarjet procedure.

This report is based on consensus-based surgical case report guidelines, SCARE criteria [7].

## 2 Case presentation

A 27-year-old male was presented with a chief complaint of deformity on his left shoulder since ten months before hospital admission. The patient slipped in a bathroom and fell in sitting position with left arm supporting the body. After the accident, the left shoulder was painful and looked deformed. Then the patient went to a bone setter and was massaged, but the shoulder was still painful and looked deformed. The patient used an arm sling to immobilize his left shoulder for about six months. Gradually, patient could do his normal daily activity with limited movement of left shoulder. Ten months after the accident, patient decided to seek medical help to treat his left shoulder.

**Fig. 1:**
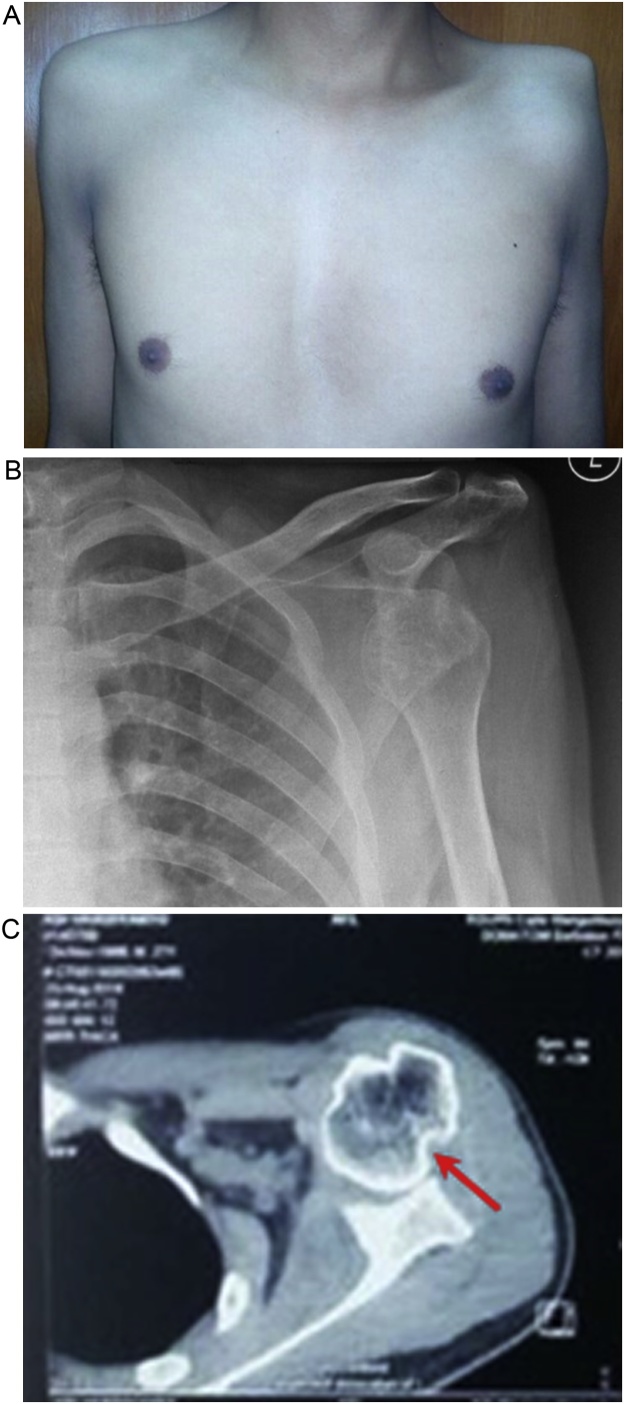
(a) Clinical picture of bilateral shoulder showing a squaring left shoulder with muscle atrophy. (b) X-ray antero-posterior view of the left shoulder showing anterior dislocation of glenohumeral joint. (c) CT scan showed a Hill-Sachs lesion on the humeral head (pointed by arrow).

The patient complained of limited movement of his left shoulder with some pain. On physical examination we found deformity on the left shoulder, liked a squaring shoulder (Fig. 1A), and muscle atrophy. Neurovascular examination was normal. The range of motion (ROM) of left shoulder was extension-flexion 20°–90°, abduction-adduction 20°–70°, internal-external rotation 30°–30°. The antero-posterior X-ray imaging showed anterior dislocation of left glenohumeral joint (Fig. 1B) and Computed Tomography (CT) scan showed a Hill-Sachs lesion on the humeral head (Fig. 1C). We diagnosed the patient had a neglected anterior shoulder dislocation with a Hill-Sachs lesion [8] and performed an open reduction and Latarjet procedure [9,10] to treat this patient.

**Fig. 2:**
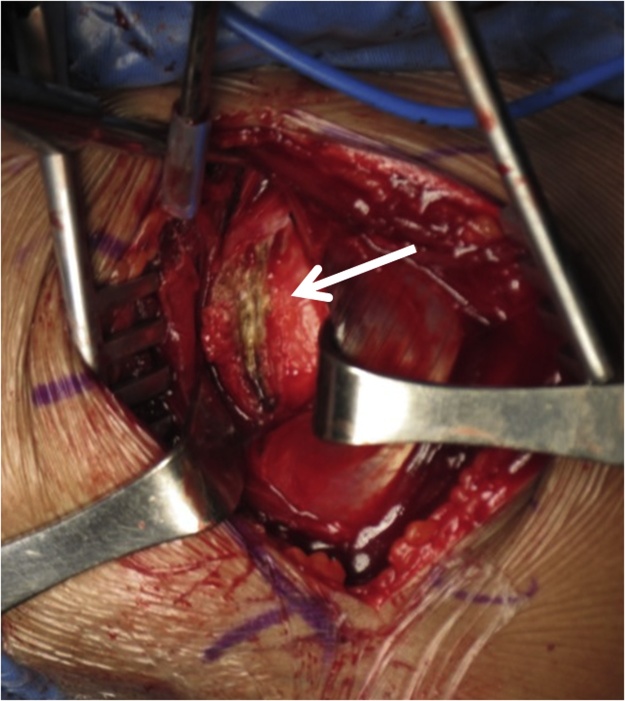
Massive fibrotic tissue around humeral head (pointed by arrow).

We performed an open reduction surgery using anterior approach of shoulder and found massive fibrotic tissue around the joint (Fig. 2) and the Hill-Sachs lesion [8]. We removed all the fibrotic tissue to create the space for shoulder joint to be reduced. After succeeded reducing the dislocation, we inserted a Kirschner wire to add stability for maintaining the reduced shoulder, then continued on Latarjet procedure. The Latarjet procedure was performed by cutting the coracoid process and transferred it with conjoint tendon to antero-posterior part of glenoid and fixed by two screws [9,10].

**Fig. 3:**
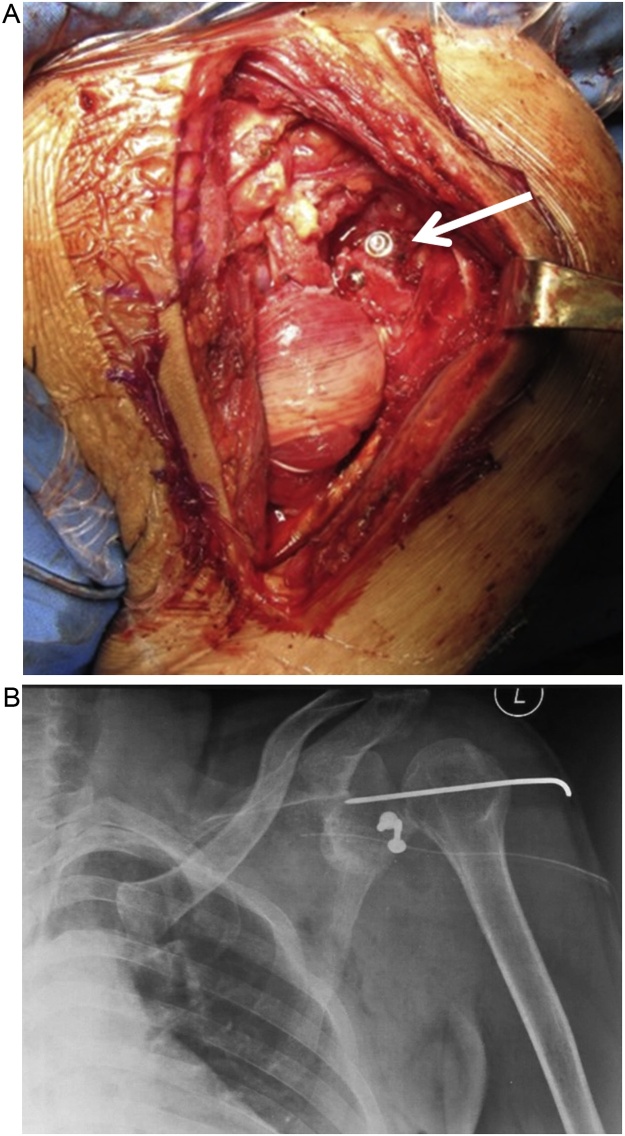
(a) Latarjet procedure: osteotomized coracoid process has been attached to antero-posterior part of the glenoid rim (pointed by arrow). (b) Post-operative X-ray antero-posterior view of the left shoulder showing a reduce glenohumeral joint with fixation of Kirschner wire and 2 screws, in accordance with Latarjet procedure.

**Fig. 4:**
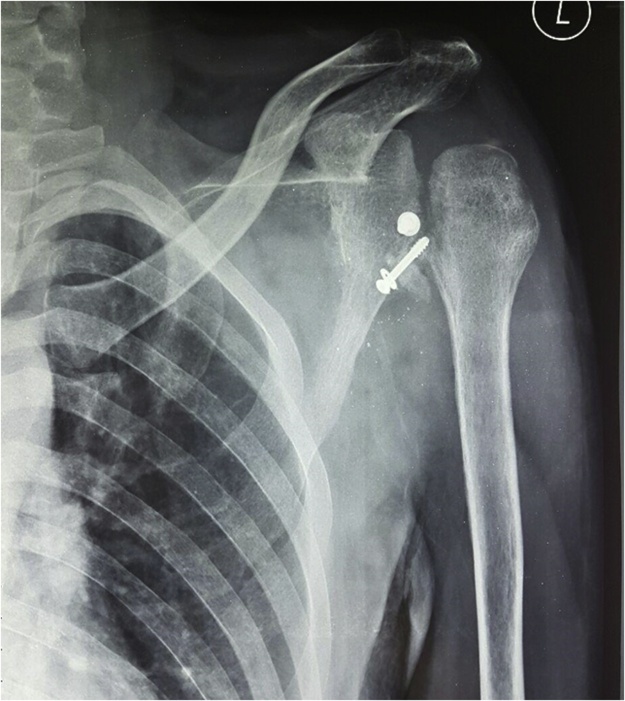
Three months post-operative X-ray antero-posterior view of the left shoulder showing loss of the humeral head contour.

The final result showed that the glenohumeral joint has been reduced with wire fixation (Fig. 3A). Post-operative X-ray showed a reduced shoulder joint (Fig. 3B). Unfortunately there was a claw hand on his left hand due to a neuropraxia of the ulna. The patient was discharged 2 days after surgery. We removed the K-wire after 3 weeks, then the patient started the rehabilitation program. The patient also underwent Transcutaneous Electrical Nerve Stimulation (TENS) and range of motion exercise for 12 times. We evaluated the patient for 3 months in outpatient clinic. Three months after surgery, the ulnar neuropraxia was healed but we found there were an osteolysis of coracoid graft and also an avascular necrosis of the humerus head (Fig. 4). The patient still had a limited ROM, (abduction 0°–100°) on his left shoulder. At ten months follow-up, the patient had no recurrent dislocation.

## 3 Discussion

The glenohumeral joint is the most frequently dislocated joint in the body [1,2]. Anterior dislocation of shoulder occurs more frequent and accounts for 95% of all shoulder dislocation [3,4]. The most common mechanism for the unilateral injuries is trauma. A traumatic event could lead to anterior shoulder dislocation when it was happened in abducted and extended arm position so greater tuberosity abuts against acromion, causing leverage forces leading humeral head to come out of glenoid cavity [2]. The term of chronic dislocation of the shoulder is applied to condition where there is loss of recognition of injury for at least 3 or 4 weeks, although other authors have described chronic dislocation with various duration [1,5,6]

A neglected shoulder dislocation may be accompanied with pathological changes in bony and soft tissue structures. Therefore, it requires an extensive surgical procedure [5,6]. A neglected shoulder dislocation, especially with signiﬁcant bony defects, is a dilemmatic condition since it cannot be managed by standard surgical procedure and concomitant lesions are common, including Hill-Sachs and Bankart lesions, massive glenoid bone loss, rotator cuff tear; and later severe glenohumeral osteoarthritis could also occur [5,6]. Because of severe soft-tissue contracture and imbalance as well as bone deﬁciency, neglected anterior shoulder dislocation is a difﬁcult problem for both patients and clinicians.

The outcomes of some procedures, such as Bankart repair, remplissage, coracoid transfer, bone-grafting and arthroplasty, in restoring the stability of the shoulder were varied and the overall failure rates were quite high [2,11,12]. The choice of treatment included observation, manipulation, open reduction with or without allograft reconstruction, Bankarts repair, capsulolabrial repair and arthroplasty [1,5,6]. Surgical treatment for chronic shoulder dislocation is usually advocated for better functional outcome, although the results may be poor and unsatisfactory [2,8]. The open reduction surgery was mostly recommended if the dislocation has been neglected more than four weeks after injury, in order to reduce the risk of concomitant fracture or cartilage injury [1,2,5,6]. Several surgical procedures have been reported a gleno-humeral transﬁxation by using smooth pins through the head into the glenoid for maintaining reduction. The acromio-humeral transﬁxing pins could halt joint motion for 3–4 weeks [1,13].

The neglected cases generally have signiﬁcant bony defects due to constant friction of the dislocated humeral head against the anterior border of glenoid, which was also found in our patient. The bony defect can cause recurrent instability but it depends on the size and depth of the defect [1,2]. In defects more than 25% but less than 40%, the anatomic procedures, such as allograft reconstruction of the head, humeral head dis-impaction/humeroplasty and non-anatomic procedures, such as osseous or soft tissue (remplissage) transfer of the infraspinatus and Latarjet procedure, are recommended. Latarjet provides stability by its ‘triple effect’ and it is more familiar for the surgeon than remplissage procedure [1,12,14,15].

Latarjet procedure has been proven to be effective for the treatment of recurrent anterior shoulder dislocation with a large glenoid osseous defect which might justify the application of this procedure for the treatment for neglected anterior shoulder dislocation. Transfer of osteotomized coracoid process into the glenoid rim was described by Latarjet in 1958. The transfer includes a portion of coracoacromial ligament which is sutured to the anterior capsule through a short horizontal incision in subscapularis. Latarjet procedure reconstructs the depth and width of the glenoid. A dynamic reinforcement is created for inferior part of the capsule through the coracobrachialis muscle, which is particularly effective when the arm is abducted and externally rotated [[14], [15], [16]]. Burkhart et al., as cited by An et al. [2], reported excellent outcome of Latarjet procedure in 102 patients, who either had more than 25% of glenoid bone loss or an engaging Hill-Sachs lesion, with only 4.9% recurrence rate after a mean follow-up of 59 months [2]. In defects that comprise more than 40%–50% of the head, rotational proximal humeral osteotomy in young patients and partial or total humeral head arthroplasty are recommended [1]. It has been suggested that, compared with soft-tissue reconstruction, such as Bankart repair, an open Latarjet procedure is more effective for the treatment of recurrent anterior shoulder dislocation with a marked glenoid osseous defect [15,[17], [18], [19], [20]]. Nevertheless, it was reported a high rate of redislocation or subluxation, loss of external rotation and internal rotation, and the deterioration or early onset of glenohumeral osteoarthritis after the Latarjet procedure [[21], [22], [23]]. In our case, the patient still had limited ROM, 100° of abduction after Latarjet procedure but no redislocation.

Nerve injury after the surgery is a common complication that can be happened during transferring the coracoid process or during surgical exploration to reduce the dislocation. The most common nerve injury is from musculocutaneous nerve and axillary nerve, but it can occur in any brachial plexus branches and mostly can be recovered spontaneously [22,23]. In this patient, the ulnar nerve neuropraxia happened during the exploration process to reduce the dislocation.

Soft-tissue imbalance is another risk factor for postoperative redislocation or subluxation. In patients with neglected anterior shoulder dislocation, the long-term dislocation may cause the lengthening and thinning of the musculotendinous unit or changing the biomechanical balance of the glenohumeral joint. A high rate of glenohumeral osteoarthritis deterioration was also noted [11,22,23]. Postoperative shoulder osteoarthritis is one of the complications that can occur due to avascular necrosis of the humeral head. There are many factors that contributing to avascular necrosis that leads to shoulder osteoarthritis for examples increased age at the time of first dislocation, increased age at the time of surgery, and presence of arthritis before surgery; however, there is no specific time when the avascular necrosis starting to occur [17]. Shoulder osteoarthritis can be occurred as a result of preexisting chondral injury, which leads to degeneration over time, or as a result of the operation procedure [20]. Despite the risk of avascular necrosis of humeral head in the long term follow up, we consider that the Latarjet procedure performed in this patient has successfully stabilized the shoulder joint of the neglected dislocation. This case report has a limitation that the follow-up period to analyze the stability of the shoulder was only 3 months after the surgery. Another long term follow-up should be considered to evaluate the shoulder stability and other surgical complications.

## 4 Conclusion

In conclusion, open reduction combined with Latarjet procedure performed for treatment of neglected anterior shoulder dislocation was found to have a high rate of successful in preventing further dislocation of the shoulder joint although high risk of osteoarthritis of the shoulder joint can still persist.

## Conflicts of interest

Andri Lubis is a consultant for Conmed Linvatec and Pfizer Indonesia.

## Sources of funding

No sponsorship for this case report.

## Ethical approval

This is a case report; therefore it did not require ethical approval from ethics committee. However, we have got permission from the patient to publish his data.

## Consent

We have written and signed informed consent obtained from the patient to publish this case report and accompanying images.

## Author’s contribution

Andri Lubis contributed in performing the surgical procedure, data collection, data analysis and writing the paper.

Muhammad Rizqi Adhi Primaputra contributed in data collection, data analysis and writing the paper.

Ismail H. Dilogo contributed in performing the surgical procedure.

## Registration of research studies

This is a case report, not a clinical study.

## Guarantor

The Guarantor is Andri M.T. Lubis, M.D., Ph.D.

## Provenance and peer review

Not commissioned externally peer reviewed.
